# Acquisition, prevalence and clearance of type-specific human papillomavirus infections in young sexually active Indian women: A community-based multicentric cohort study

**DOI:** 10.1371/journal.pone.0244242

**Published:** 2020-12-29

**Authors:** Richard Muwonge, Partha Basu, Tarik Gheit, Devasena Anantharaman, Yogesh Verma, Neerja Bhatla, Smita Joshi, Pulikottil O. Esmy, Usha Rani Reddy Poli, Anand Shah, Eric Zomawia, Surendra S. Shastri, Sharmila Pimple, Priya R. Prabhu, Sanjay Hingmire, Aruna Chiwate, Catherine Sauvaget, Eric Lucas, Sylla G. Malvi, Maqsood Siddiqi, Subha Sankaran, Thiraviam Pillai Rameshwari Ammal Kannan, Rintu Varghese, Uma Divate, Shachi Vashist, Gauravi Mishra, Radhika Jadhav, Massimo Tommasino, M. Radhakrishna Pillai, Rengaswamy Sankaranarayanan, Kasturi Jayant, for the Indian HPV vaccine study group

**Affiliations:** 1 Screening Group, Section of Early Detection and Prevention, International Agency for Research on Cancer, Lyon, France; 2 Infections and Cancer Biology Group, Section of Infections, International Agency for Research on Cancer, Lyon, France; 3 Rajiv Gandhi Centre for Biotechnology, Poojappura, Thiruvananthapuram, Kerala, India; 4 Sikkim Manipal Institute of Medical Sciences, Sikkim Manipal University, Gangtok, Sikkim, India; 5 Department of Obstetrics & Gynaecology, All India Institute of Medical Sciences, New Delhi, India; 6 Jehangir Clinical Development Centre, Jehangir Hospital Premises, Pune, India; 7 Christian Fellowship Community Health Centre, Ambillikai (near Oddanchathram), Dindigul District, Tamil Nadu, India; 8 Indian Institution of Public Health-Hyderabad, Public Health Foundation of India, Hyderabad, India; 9 Gujarat Cancer & Research Institute (GCRI), M.P. Shah Cancer Hospital, Civil Hospital Campus, Asarwa, Ahmedabad, India; 10 Civil Hospital, Aizawl, Mizoram, India; 11 Department of Health Disparities Research, Division of Cancer Prevention and Population Sciences, University of Texas M.D. Anderson Cancer Centre, Houston, TX, United States of America; 12 Department of Preventive Oncology, Tata Memorial Center, Tata Memorial Hospital & Cancer Research Inst, Parel, Mumbai, India; 13 Tata Memorial Centre Rural Cancer Project, Nargis Dutt Memorial Cancer Hospital, Barshi District Solapur, Maharashtra, India; 14 Cancer Foundation of India, Kolkata, West Bengal, India; 15 Research Triangle Institute (RTI) International India, New Delhi, India; Laboratoire National de Santé, LUXEMBOURG

## Abstract

In context of the ongoing multi-centric HPV vaccine study in India, unvaccinated married women (N = 1484) aged 18–23 years were recruited in 2012–2015 as age-matched controls to the vaccinated women and followed up yearly. We assess type-specific prevalence, natural history and potential determinants of human papillomavirus (HPV) infection in these unvaccinated women. Cervical samples were collected yearly for at least four consecutive years. A Multiplex Type-Specific E7-Based polymerase chain reaction assay was used to detect 21 HPV types.

HPV prevalence was 36.4% during 6 years. Most common HPV types were 16 (6.5%) and 31 (6.1%). Highest persistence were observed for HPV 35 (62.5%) and 52 (25%). New HPV acquisition rate was 5.6/1000 person-months of observation (PMO), highest for HPV 16 (1.1/1000 PMO). Type-specific clearance rates ranged between 2.9–5.5/100 PMO. HPV 16 and/or 18 infections were 41% (95% CI 4–63%) lower among women with 2-<3 years between marriage and first cervical sample collection compared to those with <2 years. HPV prevalence and acquisition rates in young Indian women were lower than their Western counterparts. HPV 16 infections being most common shows the importance and potential impact of HPV vaccination in India. Women with 2–3 years exposure had reduced risk possibly due to higher infections clearance.

## Introduction

A working group of the International Agency for Research on Cancer (IARC) categorized 12 types of Human Papillomavirus (HPV), phylogenetically all belonging to the alpha genus, as ‘definitely carcinogenic’ to humans [1]. These include HPV 16, 31, 33, 35, 52 and 58 (species alpha-9), HPV 18 and 45 (species alpha-7), 51 (species alpha-5), type 56 (species alpha-6), and HPV 39 and 59 (species alpha-7). Additional HPV types belonging to genus alpha (types 26, 53, 66, 68, 70, 73 and 82) have been detected in cervical cancer samples at lower frequencies and are considered ‘possible or probable high-risk’ [2]. Women with a high-risk HPV infection of the cervix have 11 times higher risk of developing high grade cervical precancers compared to non-infected women [3]. The study of prevalence of HPV (overall and type-specific) is important not only to understand the burden of infection but also to predict the risk and the incidence of HPV induced cancers in the population [4,5]. In addition, studying the prevalence of the HPV types targeted by the vaccines in young women has immense public health significance in assessing the early impact of the HPV vaccines. HPV vaccine has been introduced in the immunization programme only in two provinces (out of total 28 provinces and 9 Union-territories) in India [6]. The vaccine is expected to be introduced in rest of the country in the near future. A population-based estimate of the type-specific prevalence of HPV infection in young women from different regions of the country will create a valuable baseline for future surveillance studies assessing the vaccine impact.

IARC initiated a multi-centric study in India in 2009 to compare the efficacy of a single dose of quadrivalent HPV vaccine (Gardasil^TM^, Merck, NJ, USA) to that of two and three doses. A total of 17,729 girls received three doses, two doses or a single dose of the quadrivalent vaccine by April 2010. Follow up of the recipients of different doses of the vaccine and a cohort of age-matched unvaccinated women is ongoing, which is expected to generate valuable evidence on the long term efficacy of a single dose of the vaccine to inform the cervical cancer eliminations strategies being formulated by the World Health Organization (WHO).

The current manuscript based on the IARC HPV vaccine study reports the type-specific prevalence and natural history of 19 oncogenic (definite and probable high-risk) HPV types and two low-risk types (HPV 6 and 11, responsible for approximately 90% of genital warts) in young sexually active healthy women from seven different provinces of India. We present the data on the type-specific acquisition of new HPV infection, their persistence and clearance. Longitudinal investigation into the natural history of HPV infection allowed us to additionally identify the key determinants of incident infection.

## Methods

A total of 1540 eligible healthy married unvaccinated women, from the same rural and urban communities that the vaccinated participants belonged to were invited, of which 1484 accepted participating. The present manuscript reports HPV infection in this cohort of 1484 unvaccinated married women recruited at ages 18–23 years. The ongoing trial aims to compare HPV infection rates and incidence of cervical lesions related to HPV infection among recipients of different doses of the vaccine. The vaccinated participants received first dose of the vaccine between September 2009 and April 2010 when they were 10–18 years old and were unmarried. The unvaccinated women were recruited and provided their first cervical cell samples between April 2013 and June 2015, matched for age and study site with the then married vaccinated participants. At recruitment, the trained health workers and nurses of the study interviewed the eligible women for sociodemographic and reproductive information and took their first cervical cell samples. The health workers and nurses then visited every participant in her household every year enquiring about their general health and wellbeing. Like in the vaccinated groups, medically significant events, pregnancy, antenatal and postnatal events, delivery, and migration details were obtained and recorded through the household visits, relatives, the network of social workers, healthcare providers and hospitals records. The yearly follow up is still ongoing. The detailed study protocol and the preliminary findings on vaccine efficacy have been described elsewhere [7,8].

Cervical cell samples for HPV genotyping were collected in PreservCyt^TM^ medium (Hologic, MA, USA) from the women at their recruitment visit and yearly thereafter, until four annual samples were collected. If the fourth cervical sample was positive for any incident HPV type, an additional sample was collected to be tested for persistence. The samples were tested at the Rajiv Gandhi Centre for Biotechnology (RGCB), India, by the HPV type-specific E7 Polymerase Chain Reaction bead-based multiplex genotyping (Luminex Corporation^TM^, Texas, USA) to detect 12 high-risk (HPV 16,18,31,33,35,39,45,51,52,56,58,59), seven possible/probable high-risk (HPV 26,53,66,68,70,73,82) and two low-risk (HPV 6,11) types of HPV.

As a quality control exercise, a Global HPV DNA proficiency panel [9] provided by the International HPV Reference Center, Karolinska Institute in Stockholm, Sweden in the year 2017 was evaluated. A test was regarded as proficient in genotyping, if it could detect 50 International Units (IU)/5μl of HPV 16 and 18 DNA, and 500 genome equivalents (GE)/5μl of the other HPV types included in the panel, both in samples with single and multiple plasmids. In addition, the specificity of the reported types should be >97%.

The ethics review committees of IARC and the participating centers approved the protocol.

### Statistical analysis

The analysis of HPV genotyping, based on the cervical cell samples collected from 29 April 2013 to 10 June 2019 in the unvaccinated cohort, is reported in this manuscript. The following outcomes are evaluated:

1. Type-specific HPV period prevalence estimated from HPV types present in the participant’s first cervical cell sample (prevalent infections) and new ones detected in samples other than the first (newly acquired infections). The period prevalence reported in this analysis is based on the cumulative HPV infections accrued during 6 years of follow-up.
2. Type-specific HPV persistence defined as having the same HPV type in two consecutive samples taken at least 10 months apart. This analysis included women with at least two sample collections.
3. Type-specific newly acquired HPV infections, which involved women who tested negative for a particular HPV type in their first sample.
4. Type-specific HPV clearance defined as having a negative HPV test result for a particular type after being identified as positive for that same type in an antecendent sample from the same individual.
5. Combinations of high-risk HPV types depending on whether they may be protected, cross-protected or not protected by the vaccines (HPV 16 and/or 18; HPV 31, 33 and/or 45; and the other 14 oncogenic types) were assessed for prevalence, new acquisition and clearance, in addition to the individual types.

The effect of participants’ baseline socio-demographic characteristics and cervical sample collection patterns on any HPV infection and HPV 16/18 grouped outcomes was assessed. The sample collection patterns included three variables. The first variable was delayed sample collection. A participant was defined as having a delayed collection if she had a gap of 18 months or more between any consecutive sample collection dates. A participant who had less than four consecutive sample collections as per protocol, and whose due sample collection was delayed by more than 18 months by the date of our data analysis (10 June 2019), was also defined as having a delayed collection. The second and third variables were the number of cervical sample collections per participant, and the gap between the dates of marriage and first sample collection.

Counting of the HPV type-specific person-months of observation (PMO) for each woman in the prevalence and incidence analysis began at date of first sample collection (baseline/recruitment visit) and ended either at the date of detection of the specific HPV type of interest, or at last negative HPV test/sample collection. Women who had only one sample collection were considered to have been followed up for one day. The rates of newly acquired infections were calculated by dividing the number of new infections by the total follow-up time at risk for an individual HPV type and expressed per 1000 person-months together with their exact Poisson 95% confidence intervals (CIs). The rates were assessed based on the individual HPV type, and rates of grouped HPV infections types were also expressed per 1000 person-months, as the ratio of number of infections to the total combined follow-up time a woman was at risk of acquiring each HPV type in the respective group. The cumulative probability of acquiring a new HPV infection was assessed using Kaplan-Meier estimates of the cumulative hazard function among cohort members who were negative for a particular HPV type tested in their first sample.

PMO for clearance of each HPV type for a woman was estimated starting from the date the HPV infection was first detected to the date the woman turned negative, or to the last date the woman was detected with a persistent HPV infection for that type. Again, type-specific HPV clearance rates and associated exact Poisson 95% CIs were calculated and expressed per 100 person-months, and Kaplan-Meier estimates were obtained for the cumulative probability of HPV clearance over the study period.

The analysis to evaluate the effect of the socio-demographic factors and cervical sample collection patterns on the grouped HPV infections outcomes was carried out at the infection level rather than the woman level to increase power (i.e., an individual contributed to the analysis multiple unique HPV types acquired at different time points during follow-up). This analysis was done using generalized estimating equation (GEE) regression models to account for lack of independence between infections occurring within the same individual. All statistical analyses were carried out in Stata 15.1 (StataCorp LP, Texas, USA).

The study was approved by the International Agency for Research on Cancer (IARC) Institutional Review Board Committee on 18 February 2008 (Project No. 07–39) and by the IARC Ethics Committee on 27 March 2013 (Ref. IEC 07–39). Written informed consents were also obtained from all the recruited women.

## Results

Out of the total 1484 unvaccinated women recruited at eight study sites from seven different provinces, 229 women had one, 202 had two, 312 had three, 735 had four and six had five cervical samples assessed for HPV genotyping till June 2019. The median time of follow-up was 36.9 months (IQR: 0.03–53.0 months; range: 0.03–71.2 months). These participants had a mean age at recruitment of 20.3 years (SD: 1.1), 878 (59.2%) had high school or higher formal education and majority of them belonged to low income group. The mean age at marriage was 19 years and three-quarters had their first cervical cell sample two years or more after marriage (Table 1).

**Table 1:** Distribution of women characteristics.

Characteristics	Number of	Percentage
	Women recruited	
	(n = 1484)	
Study site and province		
Ambillikai, Tamil Nadu	200	13.5
Barshi, Maharashtra	188	12.7
Delhi, New Delhi	200	13.5
Ahmedabad, Gujarat	50	3.4
Hyderabad, Telangana	246	16.6
Pune, Maharashtra	400	27.0
Gangtok, Sikkim	100	6.7
Aizawl, Mizoram	100	6.7
Age at recruitment (years)		
18–20	712	48.0
21–23	772	52.0
Religion		
Hindu	1313	88.5
Other	171	11.5
Education		
Nil, primary or middle	606	40.8
High or college	878	59.2
Monthly household income (in rupees^a^)		
<15000	910	74.5
15000–29999	224	18.3
30000+	87	7.1
Total number of pregnancies		
None	205	13.8
1	572	38.5
2+	707	47.6
Delayed cervical sample collection^b^		
None delayed	387	26.1
At least one delayed	1097	73.9
Number of cervical cell		
samples per participant		
1–2	431	29.0
3+	1053	71.0
Gap between marriage and first		
cervical cell sample dates (years)		
<2	381	25.7
2-<3	400	27.0
3+	703	47.4

^a^ 70 rupees ≈ 1 US dollar.

^b^ A participant was defined as having a delayed sample collection date if she had gap of 18 months or more between any consecutive sample collections dates. A participant who had less than four consecutive sample collections as per protocol, and whose due sample collection was delayed by more than 18 months by the date of our data analysis (10 June 2019), was also defined as having a delayed collection.

Table 2 shows the type-specific HPV period prevalence, persistence and new HPV acquisition rate. The prevalence of any HPV infection during the 6-year study period was 36.4% (540/1484), with 19.6% (291/1484) showing infection in the first sample. The most common HPV types, either present in these women at study entry or infecting them during the study period were HPV 16, 31, 58, 56 and 68, and the corresponding proportions were 6.5%, 6.1%, 4.9%, 4.6% and 4.3%, respectively. The period prevalence was 9.3% for HPV 16 and/or 18, 9.8% for HPV 31, 33 and/or 45 and 26.0% for the other 14 types. HPV 16 and/or 18 were detected in the first sample in 3.4% (51/1484) participants. Highest persistence was observed for HPV 35, 52, 70, 16 and 18 in that order, the proportions being 62.5%, 25.0%, 25.0%, 23.6% and 22.2%, respectively. The persistence proportions were very low for both HPV 6 (6.7%) and HPV 11 (0%).

**Table 2:** Type-specific HPV prevalence, persistence and acquisition in a cohort of 1484 unvaccinated women aged 18–23 years.

HPV type	Period		Persistence^b^			New HPV infections acquisition^c^					
	prevalence^a^		No. HPV positive with			No. of	No. of	Person-	Infection rate		
	(n = 1484)		at least two cervical			women	women	months of	per 1000 PMO		
	No. of		cell sample collections			at risk	with new	observation	(95% CI)		
	Infections (%)		Total	Persistent (%)			infections	(PMO)			
**High-risk types**											
16	96	(6.5)	89	21	(23.6)	1449	61	57600	1.1	(0.8 -	1.4)
18	50	(3.4)	45	10	(22.2)	1465	31	58692	0.5	(0.4 -	0.7)
31	90	(6.1)	78	6	(7.7)	1416	22	56619	0.4	(0.2 -	0.6)
33	39	(2.6)	36	4	(11.1)	1474	29	59263	0.5	(0.3 -	0.7)
35	10	(0.7)	8	5	(62.5)	1479	5	59606	0.1	(0.0 -	0.2)
39	37	(2.5)	36	5	(13.9)	1475	28	59196	0.5	(0.3 -	0.7)
45	26	(1.8)	23	3	(13.0)	1470	12	59144	0.2	(0.1 -	0.4)
51	36	(2.4)	31	2	(6.5)	1472	24	59145	0.4	(0.3 -	0.6)
52	52	(3.5)	48	12	(25.0)	1465	33	58437	0.6	(0.4 -	0.8)
56	68	(4.6)	63	8	(12.7)	1455	39	58225	0.7	(0.5 -	0.9)
58	73	(4.9)	67	8	(11.9)	1458	47	58073	0.8	(0.6 -	1.1)
59	48	(3.2)	43	5	(11.6)	1463	27	58717	0.5	(0.3 -	0.7)
**Probably high-risk types**											
26	19	(1.3)	19	2	(10.5)	1477	12	59346	0.2	(0.1 -	0.4)
53	51	(3.4)	50	7	(14.0)	1470	37	58819	0.6	(0.4 -	0.9)
66	49	(3.3)	43	3	(7.0)	1463	28	58607	0.5	(0.3 -	0.7)
68	64	(4.3)	62	10	(16.1)	1464	44	58427	0.8	(0.5 -	1.0)
70	24	(1.6)	20	5	(25.0)	1474	14	59399	0.2	(0.1 -	0.4)
73	23	(1.5)	23	4	(17.4)	1481	20	59439	0.3	(0.2 -	0.5)
82	28	(1.9)	28	2	(7.1)	1474	18	59207	0.3	(0.2 -	0.5)
**Low-risk types**											
6	47	(3.2)	45	3	(6.7)	1448	11	58111	0.2	(0.1 -	0.3)
11	17	(1.1)	16	0	(0.0)	1482	14	59648	0.3	(0.1 -	0.4)
**Type combinations**											
16 and/or 18	138	(9.3)	126	30	(23.8)	1433	87	56696	1.5	(1.2 -	1.9)
31, 33 and/or 45	145	(9.8)	128	12	(9.4)	1394	55	55596	1.0	(0.7 -	1.3)
Other non-vaccine targeted HPV types (excluding 31, 33 and 45)^d^	386	(26.0)	351	65	(18.5)	1313	215	50132	4.3	(3.7 -	4.9)
**Any HPV type**^e^	540	(36.4)	487	94	(19.3)	1193	250	44823	5.6	(4.9 -	6.3)

HPV: Human papilloma virus.

^a^ Includes all women recruited the outcome defined as prevalent infections (present in the first cervical cell sample collection) and incident infections (acquired in cervical cell sample collections other than the first) that occurred during the study period.

^b^ Includes women positive for a particular HPV type with at least two cervical cell sample collections and the a persistent infection is defined as being infected with the same HPV type in two consecutive samples taken at least 10 months apart.

^c^ Includes women who tested negative for a particular HPV type in their first cervical sample collection.

^d^ HPV types 26, 35, 39, 51, 52, 53, 56, 58, 59, 66, 68, 70, 73 and/or 82.

^e^ HPV types 6, 11, 16, 18, 26, 31, 33, 35, 39, 45, 51, 52, 53, 56, 58, 59, 66, 68, 70, 73 and/or 82.

In women negative for any of the 21 HPV types tested on their baseline cervical cell sample collection, the acquisition rate of new HPV infections was 5.6 per 1000 PMO. The HPV type-specific acquisition rates of new infections are presented in Table 2. These rates were highest for HPV 16 (1.1/1000 PMO), followed by HPV 58 and 68 (0.8/1000 PMO each) and HPV 56 (0.7/1000 PMO each). The acquisition rates were generally higher for the individual high-risk HPV types than for the individual probable high-risk or low-risk types.

**Fig 1:**
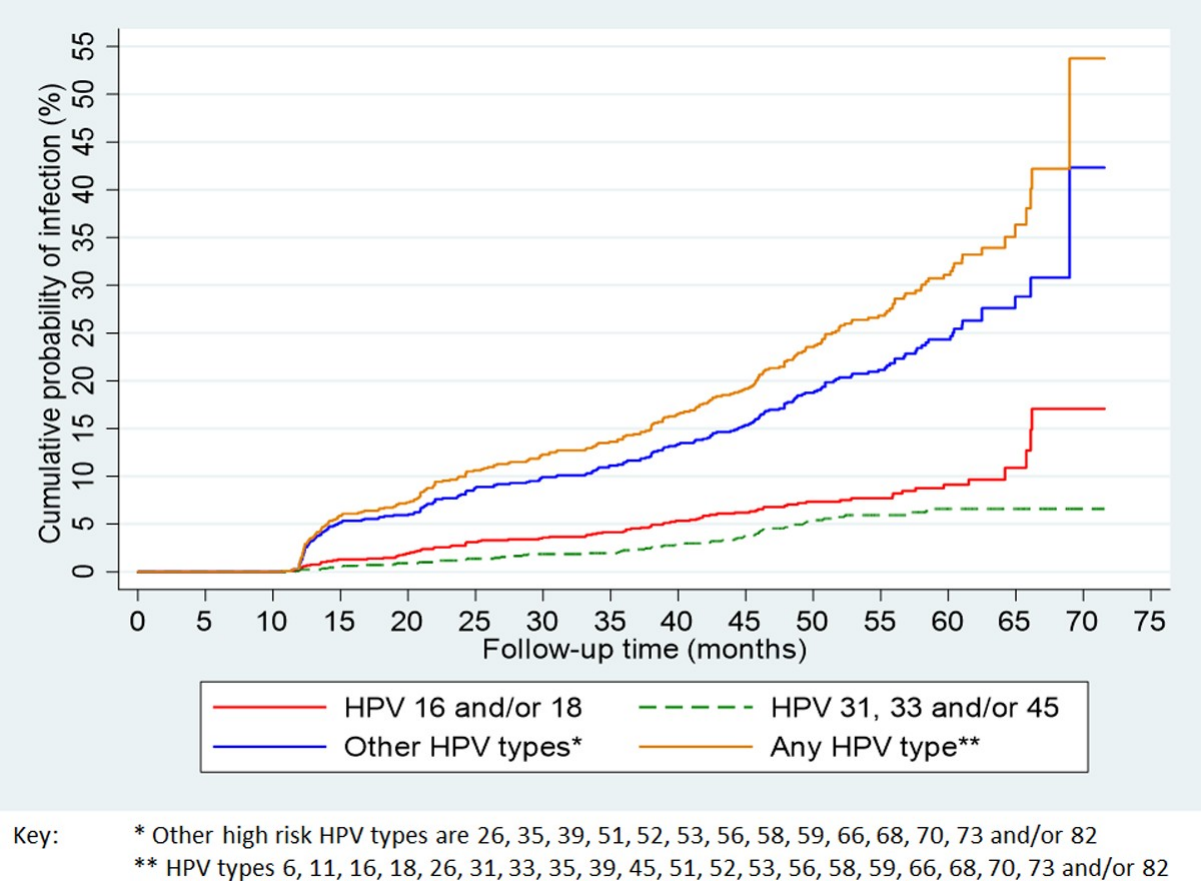
Kaplan-Meier curves of cumulative probability of HPV infection among women negative for a specific type on their first cervical cell sample collection.

The cumulative probability by follow-up time of new HPV infections was assessed for HPV 16 and/or 18, HPV 31, 33 and/or 45, high-risk types other than HPV 16/18/31/33/45 and any of the 21 HPV types (Fig 1). Women were more likely to acquire new infections of high-risk types other than HPV 16 and/or 18 or HPV 31, 33 and/or 45. After 5 years of follow-up, women had a 9.2%, 6.6% and 24.3% risk of being detected with new infections of HPV 16 and/or 18, HPV 31, 33 and/or 45 and other high-risk types, respectively.

**Fig 2:**
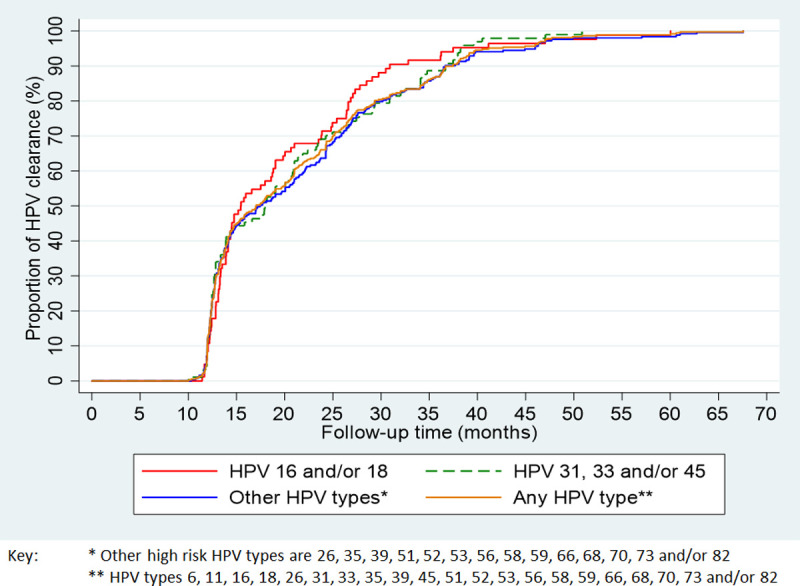
Kaplan-Meier curves of the proportion women who had type-specific HPV clearance.

Table 3 shows the type-specific clearance rates and survival time to clearance while Fig 2 presents the Kaplan-Meier plots for the cumulative proportion of participants positive for HPV 16 and/or 18, HPV 31, 33 and/or 45, or high-risk types other than HPV 16/18/31/33/45 having cleared the infection during the study period. The HPV clearance rates and proportions for the three different categories of HPV infections were similar over the course of time. The median times to HPV type-specific clearance were 1.3 years, 1.5 years and 1.5 years, and the 4-year cumulative probabilities of clearance were 97.6%, 99.0% and 97.6% for HPV 16 and/or 18, HPV 31, 33 and/or 45, and other oncogenic types, respectively. There was no significant difference in the clearance rates of the individual types, with rates ranging from 2.9 to 5.5 per 100 PMO.

**Table 3:** Type-specific HPV clearance.

HPV type	No. of	No. of	Person-	Infection clearance			Survival time to		
	women	women	months of	rate per 100 PMO			clearance (months)		
	with HPV	with cleared	observation	(95% CI)			Median (IQR)		
	infections	HPV infections^a^	(PMO)						
**High-risk types**									
16	68	55	1171	4.7	(3.5 -	6.1)	18.6	(13.3 -	26.4)
18	37	32	578	5.5	(3.8 -	7.8)	14.1	(12.4 -	23.8)
31	74	70	1475	4.7	(3.7 -	6.0)	17.4	(12.4 -	29.1)
33	18	15	297	5.1	(2.8 -	8.3)	17.9	(13.7 -	21.0)
35	7	4	138	2.9	(0.8 -	7.4)	22.1	(11.7 -	36.5)
39	24	19	389	4.9	(2.9 -	7.6)	14.7	(12.3 -	26.9)
45	19	17	345	4.9	(2.9 -	7.9)	17.9	(13.2 -	23.4)
51	21	20	396	5.1	(3.1 -	7.8)	15.4	(12.9 -	26.1)
52	41	32	702	4.6	(3.1 -	6.4)	20.8	(13.4 -	26.4)
56	40	35	808	4.3	(3.0 -	6.0)	18.8	(12.8 -	30.9)
58	53	47	1033	4.6	(3.3 -	6.1)	22.3	(13.7 -	26.4)
59	32	29	579	5.0	(3.4 -	7.2)	14.4	(12.5 -	23.2)
**Probably high-risk types**									
26	14	12	217	5.5	(2.9 -	9.7)	13.1	(12.4 -	24.3)
53	37	33	616	5.4	(3.7 -	7.5)	14.3	(12.4 -	21.8)
66	31	30	586	5.1	(3.5 -	7.3)	13.2	(12.5 -	24.3)
68	44	39	807	4.8	(3.4 -	6.6)	14.9	(12.4 -	24.3)
70	12	8	148	5.4	(2.3 -	10.7)	12.2	(11.9 -	20.1)
73	14	11	207	5.3	(2.7 -	9.5)	14.2	(12.2 -	23.7)
82	20	20	385	5.2	(3.2 -	8.0)	13.8	(12.2 -	24.6)
**Low-risk types**									
6	37	35	760	4.6	(3.2 -	6.4)	21.0	(12.1 -	26.4)
11	6	6	111	5.4	(2.0 -	11.8)	17.9	(13.6 -	20.1)
**Type combinations**									
16/18	97	84	1682	5.0	(4.0 -	6.2)	15.4	(13.1 -	25.4)
31/33/45	103	97	2031	4.8	(3.9 -	5.8)	18.0	(12.6 -	27.4)
Other non-vaccine targeted HPV types (excluding 31, 33 and 45)^b^	268	253	5503	4.6	(4.0 -	5.2)	17.4	(12.7 -	27.1)
Any HPV type^c^	387	368	7858	4.7	(4.2 -	5.2)	17.0	(12.7 -	26.9)

HPV: Human papilloma virus; PMO: Person-months of observation; CI: Confidence interval; IQR: Interquartile range.

^a^ Includes women detected with a HPV infection and found negative for that particular infection at a later date.

^b^ HPV types 26, 35, 39, 51, 52, 53, 56, 58, 59, 66, 68, 70, 73 and/or 82.

^c^ HPV types 6, 11, 16, 18, 26, 31, 33, 35, 39, 45, 51, 52, 53, 56, 58, 59, 66, 68, 70, 73 and/or 82.

The effect (obtained from the GEE regression models) of the women’s baseline socio-demographic characteristics and cervical cell sample collection patterns on the grouped HPV infection outcomes is shown in Table 4. For the HPV 16 and/or 18 outcome, a significantly elevated risk was observed in Mizoram (Adjusted Rate Ratio or ARR 6.15; 95% CI 2.32–16.26) and Sikkim (ARR 2.68; 95% CI 1.34–5.36) as compared to that of Pune, Maharashtra. No significant difference in the risk estimates was observed among the other six sites. Significantly reduced HPV 16 and/or 18 risk estimates were seen in women aged 21–23 years (ARR 0.69; 95% CI 0.48–0.99) compared to those aged 18–20 year at recruitment; those of other religious faith (ARR 0.40; 95% CI 0.20–0.81) compared to Hindus; those having 2 or more pregnancies (ARR 0.37; 95% CI 0.20–0.66) compared to the nulliparous women; those with at least one delayed cervical sample collection (ARR 0.44; 95% CI 0.26–0.73) compared to women with all collections done on schedule; participants providing at least 3 (ARR 0.55; 95% CI 0.40–0.76) compared to those providing 1–2 cervical cell sample collections; and among those whose gap between marriage and provision of the first cervical cell sample collection was 2-<3 years (ARR 0.59; 95% CI 0.37–0.96) compared to participants whose gap was <2 years.

**Table 4:** Effect of participant characteristics on overall and type-specific HPV infection.

HPV type/Characteristics	Person-	Number	Infection rate			Crude rate ratio			Adjusted^a^ rate ratio		
	months of	with	per 1000 PMO			(95% CI)			(95% CI)		
	observation	HPV	(95% CI)								
	(PMO)	infections									
**HPV 16 and/or 18 outcome**											
Overall	60,473	147	2.4	(2.1 -	2.9)						
Study site											
Ambillikai, Tamil Nadu	7,308	14	1.9	(1.0 -	3.2)	1.44	(0.75 -	2.73)	1.36	(0.71 -	2.59)
Barshi, Maharashtra	10,117	15	1.5	(0.8 -	2.4)	1.12	(0.58 -	2.14)	1.06	(0.55 -	2.05)
Delhi, New Delhi	6,821	13	1.9	(1.0 -	3.3)	1.43	(0.74 -	2.78)	0.85	(0.40 -	1.80)
Ahmedabad, Gujarat	2,270	5	2.2	(0.7 -	5.1)	1.65	(0.57 -	4.80)	0.77	(0.26 -	2.25)
Hyderabad, Telangana	6,033	13	2.2	(1.1 -	3.7)	1.62	(0.83 -	3.17)	1.12	(0.56 -	2.24)
Pune, Maharashtra	18,780	25	1.3	(0.9 -	2.0)	1.00			1.00		
Gangtok, Sikkim	4,936	36	7.3	(5.1 -	10.1)	5.46	(3.30 -	9.02)	2.68	(1.34 -	5.36)
Aizawl, Mizoram	4,208	26	6.2	(4.0 -	9.1)	4.67	(2.75 -	7.92)	6.15	(2.32 -	16.26)
Age at recruitment (years)											
18–20	30,640	80	2.6	(2.1 -	3.2)	1.00			1.00		
21–23	29,833	67	2.2	(1.7 -	2.9)	0.86	(0.62 -	1.20)	0.69	(0.48 -	0.99)
Religion											
Hindu	53,166	114	2.1	(1.8 -	2.6)	1.00			1.00		
Other	7,306	33	4.5	(3.1 -	6.3)	2.12	(1.45 -	3.09)	0.40	(0.20 -	0.81)
Education											
Nil, primary or middle	22,310	63	2.8	(2.2 -	3.6)	1.00			1.00		
High or college	38,162	84	2.2	(1.8 -	2.7)	0.78	(0.56 -	1.09)	0.78	(0.54 -	1.12)
Cumulative number of pregnancies											
at each sample collection visit											
None	7,792	41	5.3	(3.8 -	7.1)	1.00			1.00		
1	21,120	70	3.3	(2.6 -	4.2)	0.63	(0.43 -	0.91)	0.65	(0.40 -	1.05)
2+	31,560	37	1.2	(0.8 -	1.6)	0.22	(0.14 -	0.34)	0.37	(0.20 -	0.66)
Delayed cervical sample collection^b^											
None delayed	4,525	34	7.5	(5.2 -	10.5)	1.00			1.00		
At least one delayed	55,947	113	2.0	(1.7 -	2.4)	0.27	(0.18 -	0.40)	0.44	(0.26 -	0.73)
Cumulative number of cervical cell sample											
collections at each collection visit											
1–2	25,482	93	3.6	(2.9 -	4.5)	1.00			1.00		
3+	34,979	54	1.5	(1.2 -	2.0)	0.42	(0.31 -	0.58)	0.55	(0.40 -	0.76)
Gap between marriage and first											
cervical cell sample dates (years)											
<2	16,678	55	3.3	(2.5 -	4.3)	1.00			1.00		
2-<3	17,273	28	1.6	(1.1 -	2.3)	0.49	(0.30 -	0.80)	0.59	(0.37 -	0.96)
3+	26,221	62	2.4	(1.8 -	3.0)	0.72	(0.50 -	1.03)	0.94	(0.65 -	1.38)
**Any HPV type outcome**^c^											
Overall	58,852	659	11.2	(10.4 -	12.1)						
Study site											
Ambillikai, Tamil Nadu	7,190	89	12.4	(9.9 -	15.2)	1.85	(1.42 -	2.42)	1.60	(1.21 -	2.11)
Barshi, Maharashtra	9,983	64	6.4	(4.9 -	8.2)	0.96	(0.71 -	1.31)	0.85	(0.61 -	1.19)
Delhi, New Delhi	6,669	87	13.0	(10.4 -	16.1)	1.91	(1.46 -	2.51)	1.02	(0.76 -	1.39)
Ahmedabad, Gujarat	2,257	22	9.7	(6.1 -	14.8)	1.37	(0.83 -	2.27)	0.60	(0.36 -	1.01)
Hyderabad, Telangana	5,827	72	12.4	(9.7 -	15.6)	1.77	(1.31 -	2.40)	1.10	(0.80 -	1.53)
Pune, Maharashtra	18,532	128	6.9	(5.8 -	8.2)	1.00			1.00		
Gangtok, Sikkim	4,824	110	22.8	(18.7 -	27.5)	3.16	(2.50 -	3.99)	1.26	(0.94 -	1.69)
Aizawl, Mizoram	3,569	87	24.4	(19.5 -	30.1)	3.54	(2.71 -	4.64)	2.08	(1.31 -	3.29)
Age at recruitment (years)											
18–20	30,090	323	10.7	(9.6 -	12.0)	1.00			1.00		
21–23	28,762	336	11.7	(10.5 -	13.0)	1.09	(0.93 -	1.28)	0.89	(0.74 -	1.06)
Religion											
Hindu	52,262	531	10.2	(9.3 -	11.1)	1.00			1.00		
Other	6,590	128	19.4	(16.2 -	23.1)	1.89	(1.56 -	2.29)	0.72	(0.53 -	0.96)
Education											
Nil, primary or middle	21,750	268	12.3	(10.9 -	13.9)	1.00			1.00		
High or college	37,102	391	10.5	(9.5 -	11.6)	0.86	(0.74 -	1.01)	0.93	(0.78 -	1.12)
Number of pregnancies											
None	7,138	154	21.6	(18.3 -	25.3)	1.00			1.00		
1	20,652	350	16.9	(15.2 -	18.8)	0.79	(0.64 -	0.96)	0.79	(0.61 -	1.04)
2+	31,062	155	5.0	(4.2 -	5.8)	0.23	(0.19 -	0.30)	0.30	(0.23 -	0.41)
Delayed cervical sample collection^b^											
None delayed	4,461	139	31.2	(26.2 -	36.8)	1.00			1.00		
At least one delayed	54,391	520	9.6	(8.8 -	10.4)	0.32	(0.26 -	0.39)	0.48	(0.38 -	0.60)
Cumulative number of cervical cell sample											
collections at each collection visit											
1–2	24,892	441	17.7	(16.1 -	19.4)	1.00			1.00		
3+	33,948	218	6.4	(5.6 -	7.3)	0.37	(0.31 -	0.43)	0.45	(0.38 -	0.53)
Gap between marriage and first											
cervical cell sample dates (years)											
<2	16,233	211	13.0	(11.3 -	14.9)	1.00			1.00		
2-<3	16,987	160	9.4	(8.0 -	11.0)	0.73	(0.59 -	0.90)	0.86	(0.70 -	1.06)
3+	25,422	281	11.1	(9.8 -	12.4)	0.84	(0.70 -	1.01)	0.96	(0.79 -	1.17)

HPV: Human papilloma virus; PYO: Person-years of observation; CI: Confidence interval.

^a^ All variables included in the multivariate regression model.

^b^ A participant was defined as having a delayed sample collection date if she had gap of 18 months or more between any consecutive sample collections dates. A participant who had less than four consecutive sample collections as per protocol, and whose due sample collection was delayed by more than 18 months by the date of our data analysis (10 June 2019), was also defined as having a delayed collection.

^c^ HPV types 6, 11, 16, 18, 26, 31, 33, 35, 39, 45, 51, 52, 53, 56, 58, 59, 66, 68, 70, 73 and/or 82.

The risk of any HPV infection was significantly (2.08-fold) higher in Mizoram compared to Pune, Maharashtra (Table 4). There was no significant difference among the other sites.

In the quality control exercise based on the Global HPV DNA proficiency panel 2017, RGCB in-house Luminex assay was proficient for the detection of HPV 6, 11, 16, 18, 31, 33, 35, 39, 45, 51, 52, 56, 58, 59 and 68b.

## Discussion

### Main findings

Our study has systematically evaluated the prevalence and natural history of HPV infection over a median period of nearly 4 years in a young sexually active cohort of women. More than one third of the women were infected over time with any of the 21 HPV types; HPV 16 being most frequently detected followed by HPV 31, 58 and 56 in that order. Over 90% of the infected women cleared the infection by 36 months irrespective of the HPV type. The clearance rate was significantly higher in the initial months of infection–a phenomenon well-documented by previous studies [10]. This might also partly explain why women with a 2–3 year gap between marriage and first cervical cell collection had a reduced risk of HPV infection as they may have had time to clear the infection.

### Strengths and limitations

The longitudinal nature of our study helped assess HPV persistence which is the necessary cause of cervical neoplasia. This is the largest cohort so far in India in which HPV genotyping was systematically studies both for prevalence and natural history. Undoubtedly, the most important risk factors of HPV infection are related to sexual practices of the participating women and their partners. Indian society is still conservative and discussion regarding sexual practices, especially premarital or extramarital sex, is a taboo in most places. We did not collect any data on sexual practices to avoid embarrassing young women and their possible non-participation. We used gap between married and first cervical sample collection as representation of exposure time assuming marriage is a proxy for sexual debut.

Furthermore our study participants were not selected using any systematic sampling methodology and may not be truly representative of the country or the provinces they belong to. We decided not to screen this very young cohort of women for cervical cancer to avoid harms. It is possible that some of them may have had high grade cervical premalignant lesions at study entry and the data may not be exactly comparable to the outcomes of the studies that excluded such women. However, the proportion of high-grade lesions is likely to be low in these young Indian women.

Some of the persistent infections reported in our study could be due to reinfection of the cervix, which theoretically could over-estimate the rate of persistence. However, we have followed the definition of persistent infection used in majority of the natural history studies to ensure comparability of data [11].

### Interpretation

Type-specific persistence of high-risk HPV is the best known predictor of a woman developing high-grade cervical precancers in future [3]. The definition of persistence is not uniform across the studies and the reported rates vary widely with age, frequency of sample collection and number of samples collected per participant. However, there is general agreement that the high-risk types tend to persist longer than low-risk types and persistence increases significantly with age, both for low and high-risk types [12,13]. The follow-up of the unvaccinated control cohort (age 15–25 years) in a bivalent HPV vaccine evaluation study showed that more than half of the high-risk HPV infections (54.1%) persisted for longer than 1 year [14]. In our study the persistence proportions of HPV 16 (23.6%) and HPV 18 (22.2%) were higher than most of the probable high- or low-risk types but lower than that reported in the bivalent vaccine evaluation study. Another Indian study also observed higher persistence proportions for both HPV 16 (45.6%) and HPV 18 (38.4%) in 16–24 year old women [15].

The reported HPV prevalence among apparently normal women varies widely with geographical location. A meta-analysis of 194 studies conducted between the years 1995 and 2009 estimated the HPV point prevalence among more than a million women with normal cervical cytology [16]. The age-adjusted prevalence of any HPV was 11.7% worldwide–ranging from 9.4% in Asia to 21.1% in Africa. Southern Asia, almost entirely represented by studies from India, had an age adjusted prevalence of 7.1%. Another study reported significantly lower HPV prevalence among South Asian young women (<25 years of age) compared to those in Africa, Europe or America (12.9% versus 20%) [17].

HPV prevalence reported by Indian studies varied from 2.3% to 36.9%, reflecting the heterogeneity of women included in the studies [17]. High prevalence was observed in studies that recruited predominantly symptomatic women attending health facilities [18–20]. We identified only five studies from India that selected apparently normal women from the community and used a PCR based assay to detect a large number of HPV types (ranging from 26 to 44) (Table 5) [21–25]. These studies also documented a wide range of HPV prevalence, from 6.1% in south India to 19.2% among tribal women from central India. The point prevalence observed in our study (19.6%) was highest among the Indian studies possibly because of the sampling frame (which included some of the sites with high prevalence like Mizoram and Sikkim), sample collection methodology (serial samples collected from the same women over consecutive years), younger age and assay selection. The Multiplex Type-Specific E7-Based PCR assay used in our study is more sensitive than the L1 consensus primer-based PCR assays used in earlier Indian studies, and as a result significantly increased the rate of detection of HPV and the number of infections with multiple HPV types. The high analytical sensitivity of our assay has been established in a range of studies [26–28].

**Table 5:** Key outcomes of Indian studies that studied type-specific HPV prevalence using polymerase chain reaction (PCR) based assays.

Author (year)	Population selection	Study site (province)	Number tested for HPV	Test	Age	Any HPV prevalence	Most common oncogenic types (prevalence)			HPV 6/11 prevalence	
							*First*	*Second*	*Third*	*HPV 6*	*HPV 11*
Varghese et al. (2000) [20]	Community based. Women with abnormal cytology (6.7%) included	Kerala	3866	PCR with MY09/11 primers followed by hybridization with type specific probes for 27 types	15–65 years	6.1%	HPV 16 (1.8%)	HPV 33 (0.3%)	HPV 18/31 (0.2%)	0.05%	0
Franceschi et al. (2005) [21]	From rural community. Included women with abnormal cytology (4.9%)	Tamil Nadu	1891	PCR with GP5+/GP6+ primers followed by enzyme immunoassay to detect 44 types	16–59 years	16.9%	HPV 16 (3.8%)	HPV 56 (1.5%)	HPV 31 (1.2%)	0.3%	0
					<25 years	16.8%					
Datta et al. (2010) [22]	Community-based in slum areas; No cervical screening	New Delhi	1300	PCR with PGMY9/11 primers followed by reverse line blot assay for 37 types	16–24 years	11.2%	HPV 16 (3.0%)	HPV 52 (1.2%)	HPV 51 (0.8%)	0	0.1%
Srivastava et al. (2012) [23]	From post-partum clinic and from community; No cervical screening	Uttar Pradesh	2424	PCR with MY09/11 and GP5+/GP6+ primers followed by sequencing for 26 types	17–80 years	9.9%	HPV 16 (6.3%)	HPV 31 (0.7%)	HPV 33 (0.4%)	0.5%	0.2%
					< = 25	9.2%	HPV 16 (5.6%)				
Sharma et al.* (2015) [24]	Community-based among tribal women; No cervical screening	Madhya Pradesh, Chattisgarh, Jharkhand	688	PCR with MY09/11 and GP5+/GP6+ primers followed by reverse line blot assay for 37 types	18–25 years	19.2%	HPV 16 (12.2%)	HPV 18 (1.6%)	HPV 45 (0.9%)	2.6%	0.9%
Our study (2020)	Community based; No cervical screening	Eight provinces	1484	HPV type-specific E7 PCR-based multiplex genotyping for 21 types	18–23 years	19.6%	HPV 16 (6.5%)	HPV 31 (6.1%)	HPV 58 (4.9%)	3.2%	1.1%

*HPV was detected in urine samples. Demonstrated very high concordance (kappa = 0.86; 95% CI 0.79–0.94) in HPV detection from the urine samples and the cervical samples obtained from same women.

India is generally considered to be a conservative society as far as sexual practices are concerned. The National Family Health Survey (NFHS, 2015–16) collecting data to estimate the critical indicators of health reported that only 2.5% of unmarried women between 15–19 years of age had sex ever and only 0.4% of sexually active women aged between 20 and 24 years had multiple partners [29]. In spite of such apparently conservative sexual behaviour of the women, we observed that nearly one third of the study participants got infected with HPV. Aizawl (Mizoram) situated in the north-eastern part of India had the highest rate of HPV infection followed by Ambilikai (Tamil Nadu) in our study. The Family Health Survey revealed that married women in Mizoram reported the highest average number of partners (3.6%) in the country and highest HIV prevalence (1.49% compared to the national average of 0.24%). The high HPV prevalence explains the fact that Mizoram reported the highest cervical cancer incidence in the country (28/100,000 women in 2012–2014) [30].

Like our study, HPV 16 was the most prevalent type among normal women in previous studies irrespective of the study location, with prevalence varying between 2.5% in Asia and 5.8% in North America [16]. While HPV 18 has been reported to be the second most common high-risk HPV type globally, a meta-analysis of normal women [16,31] and previous studies using PCR based assays (Table 5) [21–25] in Asia observed that types such as 18, 31, 33, 51, 52, 56 and 58 could occupied the second or third positions.

The rate of acquiring a new HPV infection in our study (5.6/1000 PMO) was similar to the rate (5.0/1000 PMO) reported in another study from North India among 16–24 year old women, but much lower than that reported in the Western countries [15,32]. The 6-year follow-up of the 553 placebo recipients (age 15–25 years) of the bi-valent vaccine trials recruited in Brazil, Canada and USA observed a new HPV acquisition rate of 20.6/1000 person-months [14]. The rate of acquisition of new HPV 16 infection (1.1/1000 PMO) in our study was also lower than the rates observed in 18–34 year old Brazilian women (1.6/1000 PMO) or 18–35 year old American women (5.9/1000 PMO) [12,33].

## Conclusions

**Fig 3:**
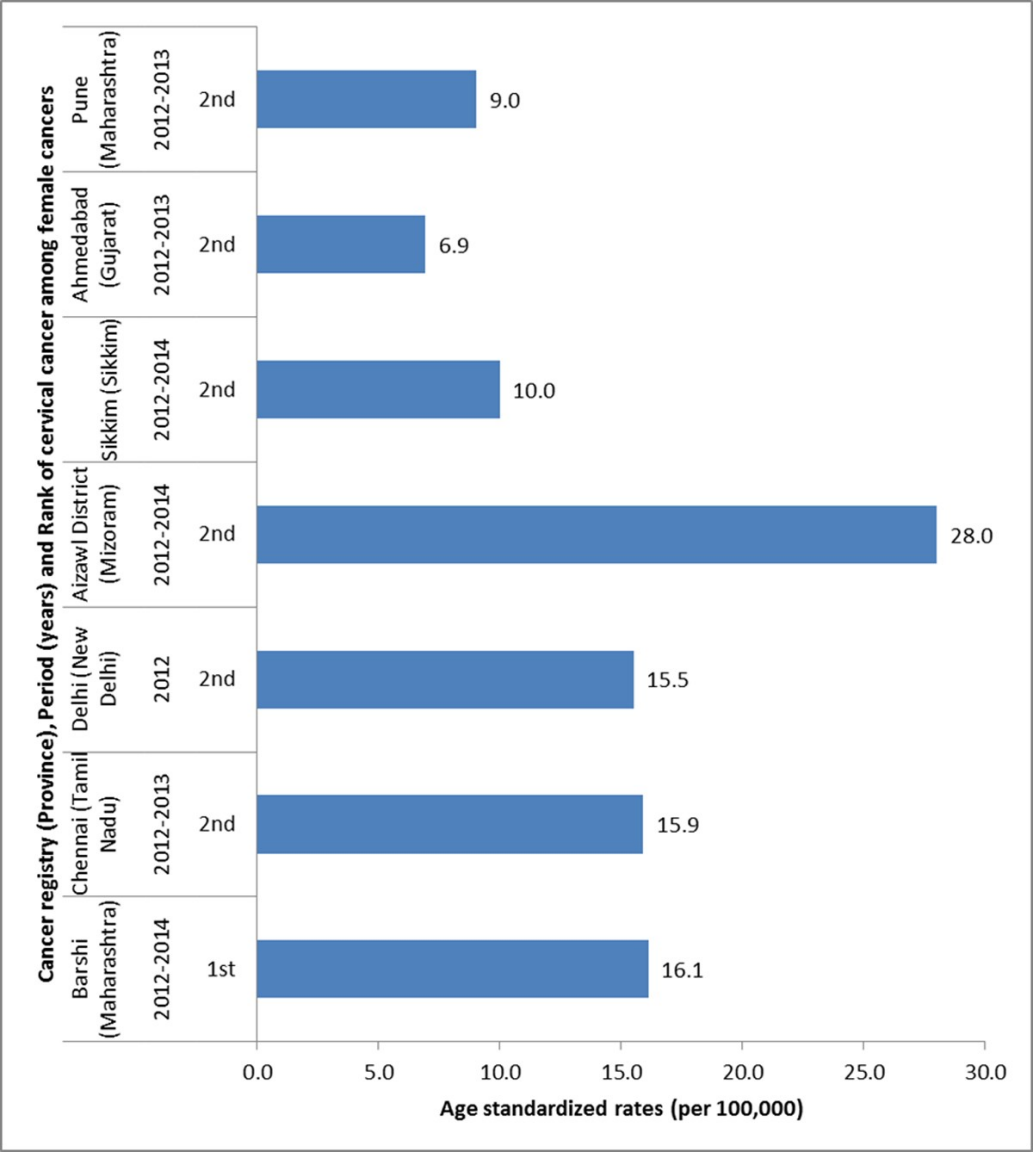
Cervical cancer incidence reported by population-based cancer registries at the Indian study sites and rank of cervical cancer by incidence among all cancers in females (Data source: National Cancer Registry Programme, India).

Indian women have a high burden of cervical cancer. The primary explanation is that women in India do not have access to population-based organized screening for cervical cancer and only a miniscule proportion of adolescent girls has been covered by HPV vaccination. Our study clearly shows that the Indian women are at high risk of being infected with HPV, especially HPV types 16 and 18. Interestingly, the center reporting highest prevalence (Mizoram) and lowest prevalence (Ahmedabad) in our study also had the highest and lowest incidence of cervical cancer among all the sites (Fig 3). Policy-makers in India should not delay introduction of the HPV vaccine any further and ensure that the women have access to quality assured cancer screening to remain on track to achieve the goal of eliminating cervical cancer.
